# Interpretive autonomy at the heart of classical musicians’ learning and well-being: the role of professional education

**DOI:** 10.3389/fpsyg.2025.1543268

**Published:** 2025-05-19

**Authors:** Marie Fujimoto, Yuri Uesaka

**Affiliations:** Department of Educational Psychology, Graduate School of Education, The University of Tokyo, Tokyo, Japan

**Keywords:** music education, interpretation, interpretive autonomy, self-regulated learning, self-determination theory, well-being, autonomy

## Abstract

A classical musician’s role is to convey their interpretations of pre-composed pieces to audiences; however, classical musicians have been criticized for conforming to normative interpretations, demonstrating a lack of autonomy in interpretation. While a lack of interpretive autonomy may harm musicians themselves by leading to maladaptive learning behaviors and lowered well-being, this has not been thoroughly examined. Additionally, interpretive autonomy may be hindered by professional training that emphasizes reproducing normative interpretations, but the extent of this remains unclear. Therefore, we conducted case study research on eight elite piano and violin students to explore how interpretive autonomy is promoted or hindered by learning experiences, and how it influences their learning behaviors and well-being. In addition, we investigated how inhibited interpretive autonomy can be promoted by education, and how earlier learning experiences in interpretation have long-term effects in college and post-college. Using a model of *Werktreue* internalization, we found that interpretive autonomy was promoted through need-supportive learning experiences, where students felt competent, autonomous, and related in interpretation; on the other hand, it was inhibited by need-thwarting learning experiences, where students felt incompetent, forced, and rejected in interpretation. We also found that interpretive autonomy is central to self-regulated learning behaviors and well-being. Furthermore, early need-thwarting experiences created psychological barriers to conveying intended interpretations during college and post-college, even when their needs were not directly threatened. In contrast, early need-supportive experiences enabled musicians to express original interpretations, even when they faced restrictive norms in the classical music field. Therefore, the study shows that while need-thwarting experiences, such as authoritarian teaching, parental overcontrol, and competitions were often implemented with good intentions to advance students’ career success, such professional education may harm their long-term artistic growth. The study also provides hope, as interpretive autonomy could be promoted by education even after being inhibited. We conclude the article with examples of learning experiences that provide students with a sense of competence, autonomy, and relatedness in musical interpretation, offering insights into how we may transform professional education for the optimal development of students and the classical music field.

## 1 Introduction

“Striving for perfection―to avoid wrong notes, wrong timbres, wrong chord progressions, wrong interpretations―often means striving to fulfill someone else’s ideal of how music should or should not sound. In this sense, anxiety about making mistakes becomes yet another mechanism for social enforcement of conformity” (Hill, 2018, p. 109).

A classical musician’s role is to convey interpretations of pre-composed pieces to audiences. Since there is no “single ideal” interpretation, musicians can cultivate their creativity and intellect in deciding what and how to communicate them, manipulating tempo, dynamics, and timbre (Palmer, 1997, p. 119; Héroux, 2016, 2018; Payne, 2016). This creative freedom results in varied performances of the same piece both within and across musicians, enriching audiences’ experiences. However, it has been argued that classical musicians have conformed to normative interpretations rather than pursuing originality, lacking autonomy in interpretation (e.g., Szigeti, 1979; Taruskin, 1995; Adorno, 2006; Leech-Wilkinson, 2020a).

To understand the complex discourse surrounding interpretive autonomy, one needs to address the *Werktreue* ideology―to be true to work―which has been regulating classical musicians’ approaches to interpretation. Around 1800, music began to be seen as artistic works by composers, and performers’ duty became to interpret works faithfully to the composer’s intentions (Goehr, 1992). In the nineteenth century, performers gradually withdrew from improvisation, as it deviated from scores, which were considered the best representation of composers’ intentions (Goehr, 1992). Early twentieth-century recordings reveal that performers still made a wide variety of interpretive choices, such as flexible tempo changes, rhythm distortions, dislocation of melody and accompaniment, added ornamentation, and use of portamento (Philip, 1992; Day, 2000). This was, however, gradually replaced by literal interpretations of notation, as greater emphasis was placed on “precision” and “clarity” (Philip, 1992, p. 233). In reviews of recordings in *Gramophone*, critics increasingly used the terms “mannerism” and “mannered” from 1951 to accuse performers of being “narcissistic” when their individuality was noticeable, acting as “the norm police” (Leech-Wilkinson, 2020b, p. 107). By the end of the twentieth century, musicologists claimed that performers refrained from conveying original interpretations to conform to the expectations within the professional community, and performances became static (e.g., Small, 1986; Taruskin, 1995; Adorno, 2006).

While a lack of interpretive autonomy may result in monotonous performances, it may also negatively affect musicians themselves. Despite their dedication, conservatory students often show various maladaptive learning behaviors which may stem from a lack of interpretive autonomy. Students who did not consider developing interpretation as part of instrumental learning followed teachers’ instructions passively (Reid, 2001) and practiced ineffectively without setting musical goals (McPherson et al., 2019). In addition, musicians who focused on technical accuracy and others’ evaluation gave unsatisfactory performances (Clark et al., 2014). In contrast, those who aimed at conveying personal interpretations displayed more effective learning behaviors, such as selectively incorporating teachers’ advice (Reid, 2001) and constructively engaging in goal setting and self-reflection in practice (Van Zijl and Sloboda, 2011; Wise et al., 2017; McPherson et al., 2019). Performers who concentrated on musical characters also entered a flow state during the performance (Clark et al., 2014).

While researchers have investigated effective learning processes by applying Zimmerman’s (2002) self-regulated learning (SRL)^1^ (see Varela et al., 2016; Dos Santos Silva and Marinho, 2025 for reviews), what differentiates effective self-regulated learners from naïve learners remains unknown. Since maladaptive learning behaviors in lessons, practice, and performance risk students’ mental and physical well-being (Perkins et al., 2017), it is urgent to identify what contributes to effective self-regulated learning. In addition, how interpretive autonomy is promoted or hindered in education remains unclear. In the absence of understanding, autonomous learning behaviors (Gaunt, 2008) and musicality or expressivity (Kingsbury, 1988; Laukka, 2004) are often attributed to students’ innate talent by teachers.

The scarcity of empirical studies on interpretive autonomy is partly due to the difficulty of defining interpretive autonomy, as definitions of what constitutes a faithful interpretation vary among musicians. Some musicians hold a subjective view where a performer develops interpretations based on subjective feelings and ideas, whereas others hold a formalist view, aiming to let the score “speak for itself” and remove oneself as a “servant” of the composer (Silverman, 2007, p. 102). While the latter may regard musicians as “subordinate” to composers (Silverman, 2007, p. 108), it does not necessarily indicate a lack of autonomy, making the issue complicated.

**Figure 1:**
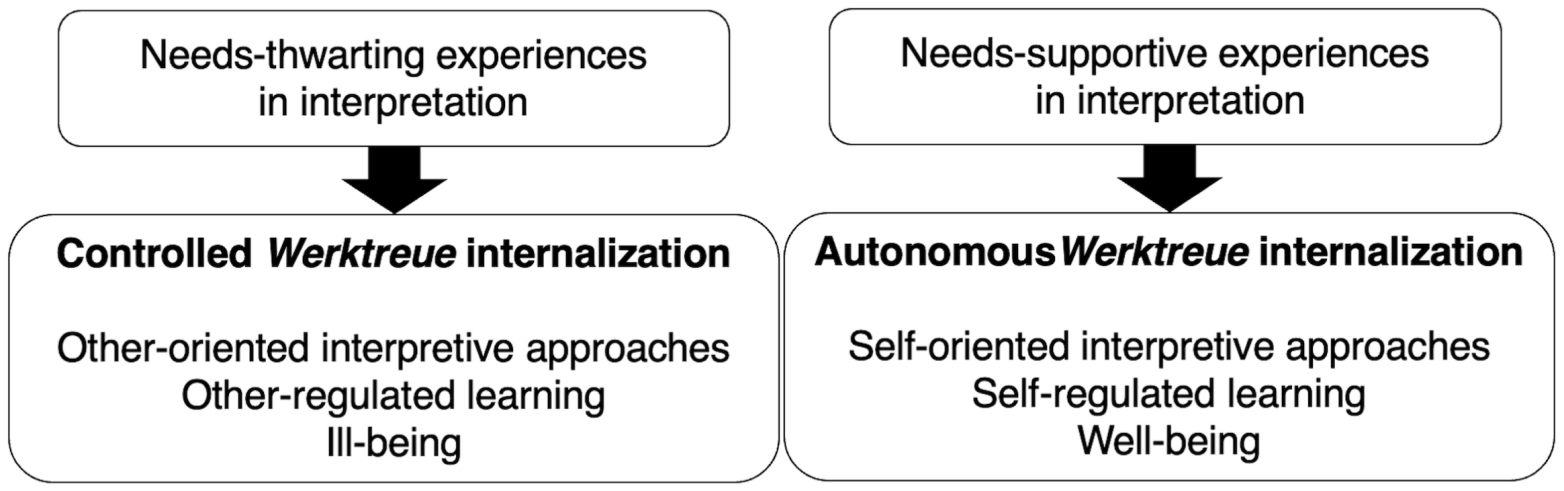
The model of *Werktreue* internalization. Adopted from Fujimoto and Uesaka (2024).

To address these gaps theoretically, Fujimoto and Uesaka (2024) proposed a model of *Werktreue* internalization (Figure 1). By applying self-determination theory (SDT) (Deci and Ryan, 2000; Ryan and Deci, 2000, 2017) to how musicians internalize the *Werktreue* ideology, the model defines interpretive autonomy, explains how musicians’ interpretive autonomy is promoted or inhibited, and shows how it affects learning behaviors and well-being.

We briefly summarize the model of *Werktreue* internalization (Fujimoto and Uesaka, 2024). When musicians’ basic psychological needs for autonomy, competence, and relatedness are supported in interpretation—meaning they perceive themselves as capable, free to make musical choices, and able to connect with others in faithful interpretation—they internalize the *Werktreue* ideology autonomously. Musicians develop interpretations based on intellectual curiosity, individual sensibilities, and values, and the ideology is integrated with the true self. This is the autonomous *Werktreue* internalization which indicates promoted interpretive autonomy. Musicians with the autonomous *Werktreue* internalization employ self-oriented interpretive approaches, which require active interpretive decision-making (Table 1). These approaches are then related to self-regulated learning behaviors which in turn enhance well-being and musical identity.

**Table 1:** Other- and self-oriented interpretive approaches.

Other-oriented interpretive approaches			Self-oriented interpretive approaches		
Approaches	Description	Examples of learning behavior	Approaches	Description	Examples of learning behavior
Impersonal	Performers restrain from imposing personal views	Failing to personally connect with music	Personal	Performers bring their personality and subjectivity into interpretations	Considering musical learning as personal development
Explicit notation	Performers follow explicit notations on a score	Failing to relate notations to musical meaning	Implicit intention	Performers neglect or change notations on a score, valuing implicit expression	Understanding musical meanings behind notations
Teacher-centered	Performers expect teachers to pass on interpretations to students	Accepting teacher’s interpretations passively	Student-centered	Performers expect students to develop their own interpretation	Evaluating teachers’ interpretation critically
Reproductive	Performers reproduce interpretations as they were rehearsed in performance	Being inflexible on stage	Improvisatory	Performers spontaneously bring new interpretations into performance	Being flexible on stage
Unconscious	Performers unconsciously develop interpretations	Lacking awareness of expressivity	Conscious	Performers consciously develop interpretations	Intentionally exploring expressivity
Separated	Performers disregard interpretations when they work on techniques	Working on segments technically without having musical aims	Integrated	Performers continually consider interpretations	Grasping an overview initially and working on techniques to express intended interpretations

Adopted from Fujimoto and Uesaka (2024).

On the other hand, when musicians’ basic psychological needs for autonomy, competence, and relatedness are thwarted in interpretation―meaning they perceive themselves as incapable, forced, and rejected by others in interpreting faithfully―musicians internalize the ideology in a controlled manner. They develop interpretations to gain external rewards, such as fame and money, or to avoid punishments, such as criticism; as a result, the ideology becomes alienated from the self. This is the controlled *Werktreue* internalization which indicates hindered interpretive autonomy. Musicians with the controlled *Werktreue* internalization employ only other-oriented interpretive approaches that are related to other-regulated learning behaviors (Table 1). These in turn lead to ill-being and disintegrated musical identity.

The model of *Werktreue* internalization also identifies learning experiences that hinder interpretive autonomy. Lessons where teachers reject students’ musical ideas (Persson, 1996; Silverman, 2008) or teach a specific interpretation as “correct” regardless of students’ individuality (Rostvall and West, 2003; Burwell, 2021) may thwart the needs for autonomy and competence. Similarly, examinations, auditions, and competitions in which students’ performances are evaluated or rejected based on pre-existing norms (McCormick, 2015) may thwart the needs for autonomy, competence, and relatedness. Conservatory students expressed anxiety about conveying original interpretations in these settings, since deviation from norms can result in being labeled a disrespectful musician by gatekeepers; yet because the norms are often ambiguous, they have to play guessing games (McCormick, 2015; Hunter and Broad, 2017; Holmgren, 2022).

Some instrumentalists, such as pianists and violinists, may be at greater risk of having their interpretive autonomy hindered from an early age especially when they aspire to become soloists. Wagner (2015) observed that children in soloist violin classes typically began playing the violin before the age of 6. Teachers then expected students to conform to “(1) technical norms, meaning technical mastery of violin; (2) attitudinal norms; behavior during the lesson and in the student’s musical interpretation and within the soloist world” (p. 103). Parents were also greatly involved, coaching their children to meet teachers’ expectations at home. Some students participated in competitions even before their teenage years with teachers’ and parents’ guidance to build a successful career.

Such early education focused on “imitation and repetition” is cautioned to undermine the long-term development of interpretive autonomy, as a “certain learned interpretation and performing style becomes their norm and sets aesthetic limits to their creative skills” (Doğantan-Dack, 2017, p. 132). Similarly, Leech-Wilkinson (2020a, chapt. 7.2) problematized how “conformist values” are ingrained in musicians’ psyches from childhood, as the teachers/parents/child form a team to achieve “the approved ways of being musical.” However, empirical research is limited.

Therefore, we conducted case study research (Yin, 2018) on elite piano and violin students to investigate how interpretive autonomy is promoted or hindered in learning experiences, and how interpretive autonomy plays a role in learning behaviors and well-being. This addresses existing knowledge gaps and validates the plausibility of the model of *Werktreue* internalization (Fujimoto and Uesaka, 2024). Additionally, we examined the chronological development of interpretive autonomy, focusing on whether interpretive autonomy could be promoted even after being hindered, and how early experiences in interpretation affect students in the long term. These are summarized below:

(1) How do need-thwarting and need-supportive learning experiences affect students’ interpretive approaches, learning behaviors, and well-being?
(2) How can interpretive autonomy be promoted even after it has been hindered?
(3) How do pre-college learning experiences affect students during and after college?

## 2 Materials and methods

Case study research is suited for investigating “a contemporary phenomenon (the “case”) in depth and within its real-world context, especially when the boundaries between phenomenon and context may not be clearly evident” (Yin, 2018, p. 15). Researchers bring *a priori* theoretical propositions to guide research design and data collection, which are supported, rejected, or modified during analysis to develop a theory that provides plausible explanations for the phenomena. The resulting theory is then considered applicable to other cases, allowing “analytic generalizations” (p. 38). In this study, we applied the model of *Werktreue* internalization (Fujimoto and Uesaka, 2024) as a theoretical framework. We also chose a multiple-case study design to identify robust findings replicated across cases.

### 2.1 Participants

Through purposive sampling, eight musicians were recruited (Table 2). All the participants were expert pianists or violinists. Seven participants had attained a Bachelor of Music at prestigious conservatories, including The Juilliard School, The Curtis Institute of Music, The Royal College of Music, The Paris Conservatory, and Seoul National University. One participant was not studying at a conservatory yet has frequently won national competitions. Participants were in their twenties (*M* = 24.88, SD = 2.17), and six of them were studying in performance programs at the time of the interviews. The nationality included three Japanese, two Chinese, one Korean, one American, and one British, and two of them were female. For privacy, we concealed the nationality and sex and translated all the quotes into American English.

**Table 2:** Summary of participant profiles.

ID	Age	Instrument	Degree	Venue	Total time (min) (time for each session)
A	24	Piano	M.M.	Zoom	175 (112, 63)
B	23	Violin	B.M.	Zoom	92
C	27	Piano	D.M.A.	Zoom	125
D	25	Violin	M.M.	Zoom	229
E	21	Piano	B.A.	In-person	309 (128, 97, 84)
F	27	Violin	A.D.	Zoom	114
G	25	Violin	B.M.	In-person, Zoom	403 (237, 166)
H	27	Piano	M.M.	Zoom	177

B.M., Bachelor of Music; B.A., Bachelor of Arts; M.M., Master of Music; D.M.A., Doctor of Musical Arts; A.D., Artist Diploma.

### 2.2 Data collection

Before the interviews, all the participants provided informed consent. The interviews were conducted on Zoom or in person in either English or Japanese and lasted from 92 to 403 min (*M* = 203 min). Three participants had multiple sessions, and each session was recorded with their permission. The first author asked questions regarding:

(1) Learning experiences in lessons, practice, and performance (e.g., How did/do your teachers teach? What was/is the practice environment like? How did/do you perceive performance settings?)
(2) Interpretive autonomy (e.g., How did/do you approach interpretation? How did/do you see current performance practices and styles? How did/do you perceive a classical musician’s role in interpretation and define the *Werktreue* ideology?)
(3) Learning behaviors and well-being in lessons, practice, and performance (e.g., How did/do you engage in lessons and feel about it? How did/do you practice and feel about it? How did/do you perform and feel about it?)

The participants freely elaborated on each question and brought other topics as they wished. After the eighth participant, diverse accounts were collected, thus data were considered adequate. Recordings were fully transcribed and used for analysis. Participants received an Amazon Gift Card equivalent to 20 dollars for their participation, and the study was approved by the Research Ethics Committee at the University of Tokyo.

### 2.3 Analysis

The first author transcribed the interviews while rendering personal data anonymous. Coding was done deductively using the model of *Werktreue* internalization as a framework. First, overarching categories were created: need-supportive/need-thwarting learning experiences, self-oriented/other-oriented interpretive approaches, self-regulated/other-regulated learning behaviors, and well-being/ill-being. All categories except interpretive approaches were further divided into three learning contexts: lessons, practice, and performance. Next, initial codes were generated under each category. For need-supportive and need-thwarting learning experiences, coding was done based on each participant’s perceived fulfillment of the basic psychological needs. When statements were unclear, the first author interpreted latent meanings. After initial codes were created, similar codes were grouped, and themes were formed. While this was not our main interest, it helped to grasp common features in learning experiences, interpretive approaches, learning behaviors, and well-being across the participants (see Supplementary Material). Then generated codes were used to organize each case to find patterns within each participant. Finally, individual cases were compared to discover common patterns across the participants.

The first researcher studied violin professionally, and this helped her to elicit and analyze detailed accounts from the participants. She also listened to the participants’ performances to deepen her understanding. Meanwhile, recognizing that her prior beliefs may affect the interview process, she asked open-ended and non-leading questions.

## 3 Results

**Figure 2:**
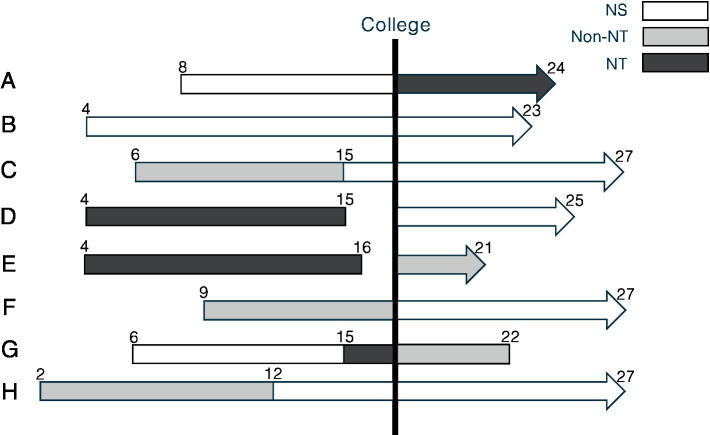
Learning experiences of the participants. The black line indicates need-thwarting experiences, the gray line indicates non-need-thwarting experiences, and the white line represents need-supportive experiences. The number indicates the age, and the black line is drawn vertically to indicate college entrance. For D and E, the lines are interrupted when they stopped taking lessons. For G, the line stopped at 22 when G left a music career.

All the participants were highly educated elite classical musicians, trained to be faithful to the composer’s intention; yet they had diverse learning experiences in interpretation (Figure 2). D, E, and G had need-thwarting experiences before college and then had non-need-thwarting^2^ or need-supportive experiences in college. Contrastingly, B, C, F, and H only had need-supportive or non-need-thwarting experiences throughout their education. Finally, A went through need-supportive experiences before college and then had need-thwarting experiences in college.

We identified four patterns that aligned with self-determination theory (Deci and Ryan, 2000; Ryan and Deci, 2000, 2017) and enriched the model of *Werktreue* internalization. While we will use limited cases to introduce each pattern, four patterns were replicated in all the cases.

### 3.1 The cause and the effects of interpretive autonomy

In this section, we will address the first research question: how do need-thwarting and need-supportive learning experiences affect students’ interpretive approaches, learning behaviors, and well-being? In short, we found that the model of *Werktreue* internalization was supported by all the cases; need-supportive learning experiences led to self-oriented interpretive approaches, self-regulated learning behaviors, and well-being, whereas need-thwarting learning experiences led to other-oriented interpretive approaches, other-regulated learning behaviors, and ill-being.

#### 3.1.1 The cause and the effects of promoted interpretive autonomy

When participants had need-supportive or non-need-thwarting learning experiences in interpretation, they adopted self-oriented interpretive approaches; they personalized interpretation, valued implicit meanings of the scores, regarded teachers as facilitators, explored interpretive ideas spontaneously, consciously developed interpretation, and regarded technique as a means for expression (Table 1). These approaches empowered participants to initiate self-regulated learning, using interpretations as “guiding tools [F]” to set musical goals and self-evaluate their own performance. They critically incorporated advice from teachers, “experimented [B]” with a wide variety of interpretive possibilities in practice, and actively engaged in performance with mastery goals to convey original interpretations to audiences. This enhanced their well-being and musical identity.

We also confirmed that self-oriented interpretive approaches are clearer indicators of interpretive autonomy than musicians’ accounts of the *Werktreue* ideology, since self-oriented interpretive approaches were adopted regardless of views ranging from the subjective to the formalist (Silverman, 2007). C expressed, “You know, they [composers] are dead. … And I just want to be able to do whatever I feel when I play music,” and, as we will illustrate bellow, C adopted self-oriented interpretive approaches. On the other hand, B had the formalist view: “My mentality is that you are a bridge between the audience and the composer. … You’re invisible on stage.” B then employed both self-oriented and other-oriented interpretive approaches: “I do a lot of spontaneous things without realizing. So, why not just focus on the things that I can put in place.” This confirms that the use of self-oriented interpretive approaches indicates interpretive autonomy (Fujimoto and Uesaka, 2024).

##### 3.1.1.1 Need-supportive learning experiences

C’s case illustrates how interpretive autonomy is promoted and how it leads to self-regulated learning behaviors and well-being.

C mostly had need-supportive learning experiences throughout their education. Starting piano at 6, C was taught by college students at a music academy. C’s parents, “not typical Asian parents,” never forced C to practice. After learning through “nothing professional” training, C was accepted into a prestigious music middle school at 12. In school, C studied with a teacher who was “too technical and philosophical,” but C did not feel limited, since C was “too young to have [C’s] own interpretation.”^3^ In high school, C had an “eye-opening” masterclass in which the teacher explicitly addressed expression and interpretation:

“I felt like [the previous teacher] never actually taught me how to express through music―how to be expressive, how to be an artist, and how I need to approach music in general. … When I got a master class from him [the next teacher], it was so fresh. Because everything was about stories, colors, imagination―all that interpretational stuff.”

After the masterclass, C switched to that teacher and studied with him throughout high school. The teacher encouraged C to develop original interpretations through open-ended questions and metaphors:

“He always asks, ‘Why do you think Brahms or Chopin wrote this way?’ … So, he would say, ‘Oh, maybe this is a conversation between a couple like a man and a woman.’ … So, he just taught me how to interpret music, I think. And how to read music―not just notes, but as a story, as a color, or as effects.”

The teacher also trained techniques so that C could express interpretive ideas convincingly:

“If there’s like a super lyrical passage, and then if I’m just playing it, he would say, ‘Oh, you should do this and that physically, so that way, you could make the phrase longer, more lyrical, and more poetic.’”

C described the teacher as “my musical godfather,” who had “the most influence on [C] as a musician”: “We had a lot of time together outside of school as well. … He taught me how to drive. He taught me how to drink. … So, basically, he taught me life.”

In graduate school, C actively chose another need-supportive teacher with whom C “literally clicked and just fell in love” during a trial lesson. The teacher accepted C’s original interpretive ideas, even when those ideas might be criticized by expert audiences:

“I mean, he loves the way I play. But also, because I have a pretty strong sense of musicality, he knows that some people would say, ‘That’s just not right’ or ‘That’s just too much.’ … And then my teacher says, ‘Oh wow, I’ve never thought of that passage that way.’”

##### 3.1.1.2 Self-oriented interpretive approaches, self-regulated learning behaviors, and well-being

C took the personal and the student-centered approaches from the beginning of the study:

“I never wanted to just obey my teacher. … I think every lesson should be something like teachers would share their interpretations or how they feel about that music. Then they could suggest many possibilities for interpreting certain passages. And students can take it if they like it. If they don’t like it, they shouldn’t take it. They don’t play for their teachers just to copy them, you know?”

C also adopted the improvisatory approach, allowing spontaneity in performance to give “the charm of live performance”: “I wouldn’t try to do like, ‘Oh, I practiced this way, so I need to do exactly this way.’ … But that’s how music is. It should be different [every time].”

C also valued implicit intentions in a score:

“I know that some people would say, ‘That’s too much’ because it’s not written on the score. … [Composers] just wrote notes and some sort of tempo markings and little descriptions, but it was never thorough. So, a pianist’s responsibility is to interpret that and then deliver it to an audience.”

Lastly, C took an integrated approach, grasping overall musical characters from sight-reading and using it as a guide when C practiced techniques: “When you see the score, you should be able to just picture what the music is trying to say. … If you don’t have an interpretation, what are you going to practice?”

Adopting self-oriented interpretive approaches, C initiated self-regulated learning effectively. When C found a need-supportive teacher who helped C to develop personal interpretation, C changed the teacher while the old teacher “did not like [C] leaving him.” In practice, C actively listened to unconventional interpretations as a source of inspiration: “They’re not confined by a traditional way of approaching classical music. So, there’s definitely a lot of freshness.” In performance, C never experienced serious performance anxiety despite being in a “very competitive” school. In addition, since C was satisfied with their own performance, C’s self-esteem was not affected by professors’ evaluations in exams:

“The thing is, even when I was not the best student there, I thought my playing was fine. And I never understood why I always got like 30 to 40 range out of 60 or 50 students. And then when I was ranked in the top three, also―I mean that’s good―but like, ‘What’s the difference?’ (laugh) … I was the same pianist.”

C pursued a music career, placing the development of original interpretation at the heart of the musicianship:

“Through my D.M.A. or the master’s [program], I’m realizing who I am as a musician. … So, my music is always about interpretation―how to make this music fresh and new or even more attractive and charming. You know, what kind of story this music has, or what kind of story I want to tell people.”

#### 3.1.2 The cause and the effects of hindered interpretive autonomy

In contrast, when basic psychological needs were thwarted in interpretation, participants employed―or were forced to employ―only other-oriented interpretive approaches; they formed interpretations that were disconnected from their personality, followed notations on scores rigidly, accepted teachers’ interpretive ideas passively, reproduced prepared interpretations on stage, were not conscious of interpretation, and practiced techniques in isolation from expression (Table 1). Taking other-oriented interpretive approaches, participants demonstrated other-regulated learning behaviors in lessons, practice, and performance; they were passive in lessons, their exploration in practice was limited, and they felt detached during the performance. This lowered their well-being; strong music performance anxiety, burnout, intake of alcohol or prescribed drugs, and physical injuries were reported. Perceiving that expressive freedom is limited in the classical music field, they became doubtful of pursuing music careers despite their high expertise and strong passion for music.

##### 3.1.2.1 Need-thwarting learning experiences

From the age of 15 to 17, G’s interpretive autonomy was severely hindered in an elite music boarding school where “everything was about perfection.”

Before then G had need-supportive experiences. G started learning the violin at 6 with warm support from parents. G took lessons with two violin teachers and “enjoyed” being exposed to different ideas: “Because I just thought ‘Oh, here are new ideas. … I can choose which one I want.’” At 8, one of the teachers introduced G to vibrato, a technique that “really attracted [G] to the violin in the first place”: “Practicing vibrato for about three hours … because I was so mesmerized by the sound that was created on the violin. … I just kept experimenting with this new technique of vibrato.” G also had frequent performance opportunities in non-music schools. When G performed with a college orchestra, G felt “an excited sensation” before the performance and enjoyed performing and seeing their delighted parents afterward. At 13, one of the teachers introduced recordings from the twentieth century, leading G to discover a musical role model, Jascha Heifetz: “I wanted to do everything like him. … He was absolutely my idol.”

Aspiring to become a musician, G auditioned and was accepted to a prestigious music boarding school at 15. Then G was assigned to a teacher, well-known for “giving students an amazing technique setup.” From the first lesson, “everything was reset.” Despite having played advanced repertoires, G was instructed to play scales and etudes for months “to reset the shape of [their] left hand” and “relearn how [G] lift and drop [their] fingers”:

“I had to completely change my bow hold because I was playing like this with my index finger pronated forward in the style of Heifetz as I admired so much. … She wouldn’t have any of it. … Vibrato was another topic that had to be completely overloaded.”

G had one-hour lessons four times a week, two of which were taught by ex-students of the teacher who were “really careful… not [to] contradict what the teacher said, especially in terms of technique.” G had no say in choosing repertoire, and the order of pieces was predetermined as each piece served “a technical purpose.” After months of etudes and scales, the first pieces G learned were Handel’s Sonatas because “they are not too demanding, and they allow you to practice a lot of technique as well.” G’s interpretation was “micromanaged” as the teacher gave detailed technical instructions, but G was never “given a reason why.” G was told that it was “better,” “the way that everyone plays today,” and “the right way to play.” Furthermore, the teacher was “so unpredictable” during the lesson:

“I didn’t know how she would react to what I was trying to do in the lesson. … She would just explode and start to raise her voice and get angry at me. To me, everything that I was doing was just trying to prevent that from happening.”

Practice was also need-thwarting, as G was instructed to follow the teacher’s instructions rigidly in case anything interfered with their technical development. Practice rooms were poorly sound-proofed, and G could “always hear other people, and there was that constant subconscious comparison going on all the time.”

One of the performance opportunities was studio class in which the teacher’s students performed one by one. Pieces became a benchmark to assess a student’s technical development, and students sounded inevitably “the same” with the same posture, fingerings, and bowings. In addition to an “extremely comparative” environment enhanced by the monotony of students, the teacher promoted criticism between students:

“I remember one person said that, after a student played through scales and etudes, they really liked their tone or something. And I just remember that [the teacher] just immediately disagreed with the student and said, ‘No.’ ‘No, I thought the tone was bad. It wasn’t good at all. Do you have ears?’ … So, everything was about negatives.”

Other performance opportunities were school concerts in which performances were, again, criticized by the teacher and judged by peers:

“[G’s peers] would only come backstage if they felt you gave a ‘good’ performance. … That level of judgment―*unspoken judgment* and comparative education where you are always being compared to or benchmarked against other students―is just not helpful. … I mean, it certainly made me feel isolated and ostracized from my fellow students [emphasis added].”

At 17, G experienced a “traumatic” performance that “left a lot of scars for years.” At that time, G started to “negotiate” over musical ideas in lessons. G then “gambled” to take a masterclass with another teacher in a summer course after obtaining permission from the teacher. In the masterclass, G enjoyed being exposed to new ideas and trying them in the “safe place.” However, when G returned to the school, the main teacher had an “allergic reaction” to the other teacher’s technical and interpretive suggestions that G had to “relearn everything again”. After two weeks, G gave “the worst performance”:

“I had successive breakdowns on stage. I had memory slips, my legs were shaking, I wasn’t breathing properly, and my hands felt completely frozen and tense. Because I was so confused mentally. … I think I was confused because I had so many ideas that I wanted to express which were put down by my teacher.”

After the performance, G was hurt by reactions from the teacher, peers, and their parent:

“Clearly, I was having serious mental problems on stage, and I did not receive the level of support that I should have received from my colleagues and from my teacher. And even my mom had no idea how to react to it. … She couldn’t understand why it happened, and she just thought that I had a problem. … Because you put so much trust into the school, so much trust into the teacher that you end up following and going along with what the school says and what the teachers say. … This was very difficult.”

##### 3.1.2.2 Other-oriented interpretive approaches, other-regulated learning behaviors, and ill-being

G never believed that “there is only one right way of doing things,” since G had been exposed to different musical ideas in childhood. However, G chose to take the teacher-centered approach, passively accepting the teacher’s interpretation “to keep my teacher happy and restful.” By reproducing the teacher’s interpretation, G also took the impersonal approach, where interpretation was detached from their own personality. G “felt emotionally numb” during performances:

“Because I just felt that I was going out on stage to demonstrate what I learned in the lesson and what I was building in terms of my technique. … And on many occasions, either the students or parents would remark that they felt very touched by my performances. But actually, I was always quite surprised that they said that because I didn’t really feel that much inside.”

G also employed the explicit notation approach: “My teacher had set all the bowings and all the fingerings for me. I couldn’t choose any of those, and even the dynamics were pre-marked. I had to play it exactly that way.” Regardless of the composer, the teacher insisted scores published by a company in a country, where the teacher was originally from. In addition, G took the reproductive approach, reproducing what was practiced on stage because “if you did something by mistake that was not what you prepared in the lesson, then that would be picked up [by the teacher].” Finally, G took the separated approach, as G was forced to go through a “disciplined systematic methodology” that trained technique in “extreme isolation.”

These other-oriented interpretive approaches prevented G from initiating goal-setting and self-evaluation. By taking the teacher-centered approach, G passively accepted the teacher’s instructions in lessons. In practice, G only practiced what was said by the teacher, and exploration was limited:

“Objective, plain practice. It was just to execute. … The moment that I felt that I wanted to express something in the old way with my own way of doing vibrato and with my own expression, the voice in my head said, ‘No, you can’t do that … because the teacher is not going to be happy.’”

In performance, G evaluated the quality of performance based on technical fluency and others’ evaluations: “So, if you didn’t have a good performance, or what they felt was a ‘good’ performance, and you didn’t execute well enough, you [were] sort of left to feel ashamed about it”.

After the traumatic performance at 17, G had “serious self-doubt and anxiety,” and G started to drink alcohol as “self-medication”: “Just to calm nervous because I was shaking so much.” G also lost their motivation to study abroad, thus G continued studying with the same teacher in college.

Although G escaped from a need-thwarting environment in college, which we will explore in the section 3. 4. 2., G decided to pursue a non-music career despite their strong passion, hard work, and high achievements:

“I eventually realized that my personality and what I wanted from music was not going to be found in the soloist’s path or career. … I disliked competitions, politics, and the level of subjectiveness [in music]. … And the thought of putting the rest of my life in the hands of other people who were choosing whether or not my music-making was worthy of management representation or competition prizes just was not for me. I wanted to be in charge of *my* future [emphasis added].”

Having gone through “pretty bumpy journey,” G wished that things were “better explained.” However, G showed great respect and appreciation for the teacher:

“I would never be able to play the violin today in the way that I can play if it wasn’t for my teacher. … That was what she felt was best for her students. … [At 15 years old] I knew that my technique was not up to the standard that was required to be a professional violinist. I was therefore willing to sacrifice my autonomy when it came to my interpretations.”

### 3.2 Promoted interpretive autonomy after being hindered

This section addresses the second research question: we found that interpretive autonomy can be nurtured even after it has been hindered.

As already shown in the case of C, participants naturally employed some self-oriented interpretive approaches from childhood, such as the personal and the student-centered approaches; even when they were unaware of the *Werkreue* ideology, they believed that music is personal and teachers are facilitators of their music-learning. G, D, and E, who had need-thwarting experiences in their teens also began to adopt self-oriented interpretive approaches once they moved away from a need-thwarting environment, resulting in improved learning behaviors and well-being. This aligns with SDT’s assumption that human beings are “active, growth-oriented organisms” (Deci and Ryan, 2000, p. 229).

While need-thwarting teachers were powerful in inhibiting interpretive autonomy, teachers were also influential in promoting interpretive autonomy. When instrumental teachers were need-supportive, all the participants started to adopt self-oriented interpretive approaches which improved learning behaviors and well-being regardless of their prior experiences. This indicates that interpretive autonomy can be nurtured even after it has been hindered, and instrumental teachers play a crucial role in it. This also aligns with SDT which posits that internalization types shift “depending on both prior experiences and current situational factors” (Ryan and Deci, 2000, p. 73).

#### 3.2.1 Hindered interpretive autonomy in childhood

In the next case, a participant’s needs were thwarted in childhood and then satisfied by a need-supportive teacher during college. The participant demonstrated significant improvement in learning behaviors and well-being.

D began learning the violin at 4 under the guidance of their mother who was deeply invested in their professional training. The mother took videos and notes in every lesson and assisted D’s practice by reinforcing the teachers’ instructions. D recalled, “I practiced about three hours around that time [at the age of 5]. But I was fighting with my mom maybe for one hour and a half. … When I was practicing a scale, she would sit by the piano and keep playing a note until I could play it in tune.” D switched teachers as D’s mother found better violin teachers. One teacher taught interpretation by talking about emotion, but it confused D. From the age of 10, D started to participate in competitions which increased their frustration:

“Before the lesson, I was like, ‘I don’t want to go to lesson today,’ and after the lesson, ‘I didn’t understand what the teacher said,’ and like, ‘I wanna stop playing.’ … And ‘I didn’t win in competitions.’”

In junior high school, D studied with a teacher, so highly sought after that other students would travel by plane to take lessons. The teacher taught how to play “inoffensive” performances that were “better technically, better for competition, but not emotionally profound”:

“[The teacher] just says, ‘OK, this needs to be more like this. … Fix this. Do this like this.’ That’s how he teaches. And very, very rarely or not even once, you would hear something emotional coming out of his mouth to influence the music. … So, it’s very, very methodical and very emotionally detached.”

At 13, D took the impersonal approach, the unconscious, and the teacher-centered approaches:

“I always played with some kind of musical phrasings intuitively. … But I was emotionally detached. … When I was studying with the teacher, I really hated playing the violin, and I don’t think any emotion truly came from my heart when I was playing.”

This made D passive in lessons: “I had no opinion. I just stood in the lesson room and did whatever I was told. … I was like, ‘I will do it if you tell me what to do.’” D never practiced voluntarily. In performance, D often compared their own performances to those of older students, which lowered D’s self-esteem: “I thought ‘I can’t play like them. I’m a failure.’” Being “burned out,” D stopped playing the violin for about four years.

#### 3.2.2 Promoted interpretive autonomy in college

D then entered a college which housed a conservatory. D’s mother asked to have a trial lesson with a violin faculty at the conservatory, and this became a turning point:

“During the lesson, I told him, ‘I don’t like this competitive music world where you are always compared to others.’ … Then he said, ‘The point of music is not competition. … The most important thing in music is that you keep pursuing your own performance.’ I was really moved by his words. I thought, ‘For that then, I can work hard―not to win, but to strive for more beautiful performances.’”

D auditioned for a conservatory to study with the teacher. In lessons, the teacher warmly accepted D’s musical ideas and valued their long-term development:

“When I introduced my musical ideas, he never rejected them. … Also, when I was concerned about my life, he was very supportive. So, I felt that he cared not only about my violin studies but also my personal development. … He didn’t pressure me to enter competitions. Instead, he focused on helping me connect my emotions with the music while training my techniques.”

This led D to take self-oriented interpretive approaches: “I realized that I have to have a good reason for what I do.” Then D started to demonstrate effective learning behaviors. In the lessons, D “asked a lot of questions”: “I often said, ‘I want to make this kind of sound, but I can’t,’ and ‘I want to express this, but I don’t know how.’” D practiced five to six hours every day:

“So, after studying with him for a while, I began to reflect more on my emotions in practice. … For the first time, I discovered the joy of pursuing emotional meanings in music.”

In performance, D initially struggled with anxiety, being preoccupied with negative thoughts. However, toward the end of college, D focused more on conveying personal meanings:

“At conservatory, I started to become aware of how, if I don’t perform ‘well,’ I am getting judged by my colleagues … It influences who asks you to play together or what opportunities you get. A conductor might be watching, and he might not put you in the first chair, if he hears you play badly in public, right? … And then what if other faculties happen to be there because they have the power to give you performance opportunities, and you might not get them, right? … So, the fear of getting judged and the consequences of that holds. … That was a very fundamental fear. … [By the end of college] a little bit that started to shift toward ‘Can I be convincing? Can I connect with the audience? Can I show who I am through my music?’”

After college, D auditioned a prestigious conservatory for a graduate program to study with another teacher who helped D achieve a personal goal “to use [their] body more naturally” to connect with music. After graduation, D pursued a music career, recognizing that music provides the most meaningful experiences for D.

### 3.3 Interpretive autonomy as resilience against need-thwarting environments

While this was not part of our research questions, we found that interpretive autonomy enhanced musicians’ resilience in need-thwarting environments. When participants with self-oriented interpretive approaches perceived that their needs were being thwarted by authoritarian teaching, they tried to change the teacher or school. When changing the environment was not possible, they engaged in alternative activities to fulfill their needs, such as freely exploring interpretive possibilities in practice or listening to unconventional interpretations. While this resilience was evident both in childhood and college, as we will illustrate, children were more susceptible to need-thwarting environments.

In contrast, while other-oriented interpretive approaches were often employed to adapt to a need-thwarting environment, these approaches made their learning more dependent on others, increasing their vulnerability to such environments. In addition, to improve the situation, participants reinforced other-regulated learning behaviors, such as following a teacher’s instructions rigidly, which thwarted their needs further as illustrated in G’s case above.

This indicates that interpretive autonomy, promoted by need-supportive experiences, enhances need-satisfaction further, whereas a lack of interpretive autonomy, forestalled by need-thwarting experiences, thwarts the needs further. SDT supports this, as while “autonomous regulation involves greater need satisfaction” (Deci and Ryan, 2000, p. 243), controlled regulation that “serve[s] to protect them from the threat and preserve as much satisfaction as seems possible in the non-supportive situations … has the unfortunate consequence of continuing to thwart need satisfaction, even in situations where satisfaction might be available” (p. 249).

#### 3.3.1 Resilience in college

We will present two cases of participants who demonstrated strong resilience against need-thwarting environments in college and childhood. While A, having gone through need-supportive experiences beforehand, demonstrated a strong resilience in college, E’s resilience is threatened due to enduring need-thwarting environments from childhood.

A mostly had need-supportive learning experiences until college. A began learning the piano at 4 “casually” with their father who never pressured A to practice. A also studied with need-supportive teachers who often praised A’s interpretation that it “tell[s] a story.” A enjoyed performing in public, whether in competitions or studio classes.

In college, however, A studied with a need-thwarting professor who constantly criticized A’s interpretations for being too personal for six years:

“She always keeps telling me, ‘Your playing is always yourself too much. … You just play what you feel.’ … When I was playing Scriabin’s Sonata, she said ‘What you are playing is [A]’s Sonata. Not Scriabin’s Sonata.’ … She sometimes comments like, ‘Your playing is in general like a primitive person.’”

Furthermore, the teacher did not demonstrate on the piano but would “sing very ugly, imitating what [A] played,” so A was confused and frustrated.

Being always “yelled at,” A got nervous before the lessons. One day before a graduation recital, the teacher told A that their playing worsened compared to five years ago, making A self-doubt, “Whether I really play like that or not.”

However, A showed strong resilience against the teacher, as A believed that interpretation is personal and students should develop their own interpretations:

“It’s easier to think that way actually … [to play] to make her happy otherwise she will kill [you]. It’s very easy to think that way, but I don’t think like that. Because no matter how well you play, she would still kill you. (laugh) … I think her attitude is right. You have to study the score very carefully because that’s the only reference the composer left. But how do you know your impression of this music is right, and my impression is not right?”

A participated in competitions without consulting with the teacher, saying, “because if I asked, I know she will say, ‘No.’” While the teacher told A to find a pre-composed cadenza by others, A composed their own cadenza. During performances, A forget about the teacher and focus on the music, adopting an improvisatory approach: “Because what [the teacher] says is always very detailed. If I think of too many details, I can’t really play. … You will never catch up with music.”

A pursued a music career with a clear artistic goal, and A recalled a recent performance of performing a favorite piece from childhood:

“After I finished, [the audience] all came to ask me, ‘What’s the title of that piece again? Can you say that again?’ … Actually, it makes me very happy. Since that’s actually one of my dreams to spread all [A’s national] music to people all over the world. … Today, I think I just let them understand better what [A’s national] music sounds like.”

#### 3.3.2 Resilience in childhood

In the case shown above, the participant had need-supportive experiences before college. In the next case, however, E mostly had need-thwarting environments from the beginning of the study. While E initiated SRL with self-oriented interpretive approaches, E gradually suffered from need-thwarting learning experiences.

Having started piano lessons at 4, E participated in competitions every year from the age of 5. The first teacher greatly cared about results in competitions, and E’s parent took videos and notes during lessons and supervised practice at home. Before competitions, parents―both of them could play the piano―assisted E’s practice, sometimes all day.

While these learning experiences could be perceived as need-thwarting, E appreciated the teacher for teaching “the enjoyment of music.” E also thanked the father even though E “cried badly” when E negotiated musical ideas with him:

“Because my father didn’t give up, I had to accept his ideas. … So, when I accepted it and tried his musical idea, I thought, ‘It’s actually good!’ Then I didn’t feel like my musical ideas were put down. …. And I felt like I improved a bit. … I was probably around 8 years old.”

E was resilient because E was always interested in learning composers’ intentions and felt that they helped E to understand music better. When E discovered a favorite composer Chopin at 8, E composed pieces in the style of Chopin. E enjoyed reading composers’ biographies, finding the composers relatable.

Taking the personal approach, E engaged in various musical activities that satisfied their needs in interpretation. In practice, E enjoyed sight-reading a variety of pieces other than assigned pieces and listening to favorite interpretations. In junior high school, E played the viola in an ensemble club and accompanied a choir. In high school, E organized and performed two recitals with friends in a local town.

However, E gradually struggled with need-thwarting environments which pressured E to adopt other-oriented interpretive approaches. While E won the first prize several times in competitions, the preparation eventually became overwhelming for their parents. At 9, they switched to a teacher who disliked competitions. The teacher constantly rejected E’s ideas and did not allow E to choose pieces or musical ideas that would be beneficial in competitions. E, nevertheless, respected the teacher’s values and adopted the teacher-centered approach:

“When I tried to make the music more interesting, the teacher rejected my ideas. He was quite the opposite of my first teacher, and when I played with obvious expressions, he said, ‘It’s unnatural.’ … In every lesson, I did my best to understand what he wanted, though.”

At 13, E was eliminated in a competition:

“It was shocking because I listened to other performances, and I didn’t like them. … Yes, I did make some mistakes, but I was confident that my music was better.”

Because the previous teacher’s student advanced to the next round despite her “uninteresting” performance, E returned to the first teacher and won a prize a year later.

In high school, E gave an improvisatory performance that was “full of musical ideas but no control,” which “just exploded” in a competition. E was eliminated from the round and advised to play more stably by a conservatory professor. In the final year of high school, E decided to apply to an academic university that has no music performance department: “I wanted to keep the feeling from my childhood that music is wonderful. I thought, ‘If I go to a conservatory, I will lose myself because I have to compete with others.’”

### 3.4 The long-term effects of early learning experiences

The last section addresses the third research question: how do pre-college learning experiences affect students during and after college? Analysis revealed that earlier learning experiences had lingering effects on students when they began to develop their interpretations actively during and after college. Participants who had need-supportive learning experiences earlier could focus on conveying original interpretation in performance even when they felt restricted by interpretive norms within the classical music field. In contrast, those whose needs were thwarted earlier experienced anxiety and a sense of detachment in performance when they tried to convey their interpretation.

#### 3.4.1 The effects of early need-supportive learning experiences

Participants with earlier need-supportive learning experiences demonstrated strong resilience against need-thwarting environments. This is already introduced in A’s case, who resisted the teacher’s criticisms, but it was also apparent when the need-thwarting figure was more conceptual.

In college, six out of eight participants recognized the classical music field as need-thwarting; they were aware of perfectionistic standards in interpretation, limited interpretive choices, and intense competition among peers to present “better” interpretations. The six participants also recognized and criticized normative interpretations, describing them as “lazy [F],” “boring [C, D, G],” “plain [C],” “tiring [G],” “dangerous trend [A],” and “unnatural [E].” Their authenticity was also questioned:

“Very accurate with the music, technical perfection, and not very improvisatory. That’s like the standard, right? … Mozart, I think it should be a lot more free than how a lot of people play it. Because he himself was very improvisatory. … A lot of contemporary pianists don’t [improvise]. Because they are afraid to, and they don’t know how to [D].”

They also noted that audiences “hate [C]” musicians who present atypical interpretations and even world-renowned musicians did not necessarily convey unique interpretations.

Despite such a situation, earlier need-supportive experiences let students focus on conveying their original interpretations, regarding it as a crucial role of classical musicians. C recognized restrictive norms in competitions and the field in general, noting that there is “definitely a very favoured interpretation”:

“Like very virtuosic and dramatic, but nothing too out there. Nothing too crazy. … And then if you can fit yourself into those criteria, you are considered a good player. And if you do like excessively rubatoes, or if you do dynamics too intensively, people will say, ‘Oh, that’s just not right.’”

Then C modified the *Werktreue* ideology in a way that freed C from interpretive norms:

“I try to refuse already pre-existing connotations of composers that ‘Bach should be this way’ … because we really don’t know who they were in real life. … So, I think there are limitless possibilities. … So, I try to bring a fresh, new perspective of each composer or each piece.”

Similarly, having studied with teachers who were “all about, even if you don’t get a good result [in competitions], you should stay true to yourself,” F stated that without developing personal interpretation, “it loses the meaning of playing written music completely.” F also modified the *Werktreue* ideology effectively, allowing themselves to be driven by the goal of making an impact on audiences through the music:

“You revive the feeling that the piece, or the impact that the piece created at the time for the listeners back then. … When Beethoven’s 9th Sonata was performed, it was artistic terrorism to the listeners at the time. And then for this sonata to create the same impact for modern audiences, it needs to be much more extreme for people to feel like ‘This is terrorism. This is not music.’ … The composer’s intentions are important, but they are important, not because you want to play exactly how it was played back then.”

F also recognized restrictive interpretive norms but managed to convey intended interpretations. Before a competition, F was told by a teacher, “I really like how you played this, but I know people on the jury are going to have problems with it.” F then felt, “If I go into a competition with too much of my own thoughts or what I think of the music, I would get washed out.” F initially tried to make interpretive decisions “less aggressive” and “less unheard of,” not being “my true self because it was a toned-down version of it.” However, it was “really difficult” to assimilate into a preferred interpretation, so F changed the strategy “to choose repertoire that [F’s] idea sort of lines up with what general people think.” This led F to be more comfortable: “So, going in [the competition], I didn’t really think about ‘Oh, I have to play this way or that way.’” In recital, F is focused on sharing their “vision of what the music sounds like” and has done creative projects to “mak[e] this a real experience … instead of putting classical music on a high horse, and we think of ourselves as highly educated elite.”

The remaining two students who had only need-supportive experiences did not perceive the classical music industry as need-thwarting, unlike the other six participants. This may be due to their positive experiences with normative performances during their teenage years. H was often moved by “beautiful” performances in lessons and concerts: “I was so touched by their performances. They made me think, ‘This is what music is.’” H did not recognize interpretive norms because H “listen[s] to others’ performances to get inspiration, not to evaluate if they are playing ‘correctly.’” H has been actively engaged with solo and chamber concerts to “deliver love through music to people as much as possible.” B recognized that “in very famous pieces people just do the same kinds of things,” yet B stated that “individuality is overrated” in the field, reflecting their formalist view that performers should “remove [themselves] from the playing.” B recalled that B’s teacher was “always thinking about the composer first, and that’s what ma[de] his playing so special.”

Interestingly, accepting the formalist view was not so straightforward for B; B wanted to be “unique” and “special” but adopted the formalist view during college years due to a sense of “failure”:

“I didn’t understand that you have to remove yourself from the playing. … If you play in concerts, you get the sense of what the audience *actually* thinks based on what they’re saying. … You shouldn’t force trying to be unique. … If you force it, it’s bad because people can tell. … I think my biggest pet peeve is people that try to show off. So basically, I’m the biggest pet peeve myself from two years ago [emphasis added]”.

B frequently emphasized that B’s view is yet “always changing”: “I’m OK not sounding up to my full potential right now as long as I’m trying things. … I think once I find my voice, I’m going to also establish a taste in music a little bit firmer.”

#### 3.4.2 The effects of early need-thwarting learning experiences

In contrast, early need-thwarting learning experiences had negative long-term effects; participants whose interpretive autonomy was hindered earlier struggled to convey their original interpretations in performance. They often experienced strong music performance anxiety beforehand, felt disconnected from the music during the performance, and were dissatisfied afterward, even when their needs were no longer thwarted directly. This was particularly evident in performance settings where interpretive norms were shared among audience members, such as school concerts, auditions, and competitions.

After two years of need-thwarting learning experiences, G escaped from the aversive learning environment in college. The same teacher gave no more need-thwarting lessons, treating G as an “adult.” G was at a higher level than most students, and G won a prize at an international competition. Naturally, G took self-oriented interpretive approaches: “I wasn’t thinking in terms of what would my teacher like. … I just wanted to achieve quality in my playing with as much individuality as I could manage. … I would just come to the lesson with my interpretation.”

However, even then G suffered from anxiety when G tried to convey personal interpretation in performance. G recalled the competition:

“It’s not that I deliberately played in a way that I thought [judges] would like, but I just remember feeling very self-conscious about whether what I was doing was correct or what I was doing was in good taste or acceptable. I found it quite hard to be myself.”

Before lessons and performances, G continued to drink alcohol as “self-medication,” which was eventually replaced by prescribed beta-blockers. This is because the abovementioned performance at 17 gave “PTSD” that “every occasion that [G] stood up and played the violin in front of someone, it was a trial or a battle.” Despite G’s technical ability was “only getting better,” G’s mentality was “seriously getting behind,” making G decide to pursue a non-music career. Even when G started studying a new field, G suffered from a perfectionistic “insidious mentality” and was “learning to shake all of that off.”

Similarly, while D’s learning behaviors improved drastically during college, D recalled struggling with performance anxiety in graduate school. D once had a mental breakdown before studio class because “emotionally [D] wasn’t able to connect with [their] music”:

“I had been struggling with that for like a month. … I was already about to burst into tears by the time I started because I was like, ‘I know I’m not emotionally there, and I don’t know how to reconnect with my playing emotionally.’ … And it didn’t go well. And then I ended up crying after. Then [the teacher] was like, ‘Oh my gosh,’ and everyone was so nice though. But that kind of specific struggles that I was having with music would sometimes be a source of nerves.”

Lastly, even though E naturally exhibited strong interpretive autonomy from childhood, E expressed discomfort in conveying interpretations in lessons and competitions:

“I feel like I’m trying too hard. … In competitions, I suddenly made mistakes on stage, and I was always concerned with that. But I didn’t know what to do with it.”

E was also aware that their interpretation conflicted with their father’s ideas:

“When I was in elementary school, I couldn’t really explain the differences since my ideas were not crystallized as much as his ideas, but I certainly felt that they were different. … My father wants to hear music that convinces him. He believes that if I play in a way that satisfies him, I will achieve good results—and he’s probably right. But neither of us can forgive ourselves for not playing the way we truly want to. And I don’t want any trouble from this.”

While recognizing the restrictions on interpretation in competitions, E continued participating in competitions: “Since I am not at conservatory, I think winning competitions makes it more socially acceptable for me to continue playing the piano. It might open up career and performance opportunities too.” E continued:

“I just want to find a way out. … When I was in high school, I thought if I became a professional, I would be stressed out. … But now, I’d accept it if I could continue living with music. It’s definitely better if I could focus only on music in my life, right?”

### 3.5 Summary

Four patterns were found that supported and enriched the model of *Werktreue* internalization (Fujimoto and Uesaka, 2024). First, need-supportive learning experiences in interpretation led the participants to adopt self-oriented interpretive approaches regardless of their varied stances on what constitutes an authentic interpretation. With self-oriented interpretive approaches, participants initiated self-regulated learning, maintained well-being, and pursued music careers with integrated identity as classical musicians. In contrast, need-thwarting experiences in interpretation forced participants to take only-oriented interpretive approaches regardless of their ability to develop interpretation. They then showed dependent learning behaviors, lowered well-being, and doubt about pursuing a music career.

Second, all the participants showed a natural inclination toward interpretive autonomy; even when their interpretive autonomy was hindered, once they moved away from a need-thwarting environment, they started to take self-oriented interpretive approaches. Instrumental teachers were especially influential in supporting such orientation toward growth. This indicates that interpretive autonomy can be promoted even after it has been inhibited, and instrumental teachers were powerful in supporting the participants’ needs in interpretation.

Third, while this was not part of research questions, interpretive autonomy buffered the negative effects of need-thwarting learning environment. Participants adopting self-oriented interpretive approaches could successfully manage the aversive environments, whereas participants with other-oriented interpretive approaches were more vulnerable to the environment. Additionally, the participants were more susceptible to the need-thwarting environment in childhood even when they showed a strong inclination toward interpretive autonomy as a child.

Lastly, learning experiences in interpretation before college had long-term effects on musicians in college and post-college. Participants with earlier need-supportive learning experiences could perform their original interpretations satisfactorily even when they perceived restrictive norms within the classical music field. In contrast, participants with need-thwarting learning experiences struggled psychologically when they tried to perform their personal interpretations. This difficulty persisted even when their needs were not directly threatened by the environment and was particularly evident in evaluative performance settings, such as competitions.

## 4 Discussion

These findings show that (1) interpretive autonomy supports students’ self-regulated learning and well-being in both the short- and long-term, and (2) it is essential to support students’ interpretive autonomy from the first stage of learning.

### 4.1 How does interpretive autonomy support students’ learning behaviors and well-being?

While the importance of interpretive autonomy has been implied for instrumental learning (e.g., Reid, 2001; Clark et al., 2014; McPherson et al., 2019), this is the first empirical study that revealed a close relationship between interpretive autonomy, SRL, and well-being. Interpretive autonomy was essential in promoting SRL and well-being; to develop and convey original interpretation, students effectively adopted teachers’ advice, engaged in explorative practice, and were fully focused on conveying intended interpretation in performance. This led to enhanced self-efficacy and integrated identity as classical musicians. SRL also promoted interpretive autonomy; for example, adopting a teacher’s advice selectively satisfied the need for autonomy in interpretation, showing a mutual relationship.

Interpretive autonomy especially helped the participants to initiate SRL in performance. Even in high-stake performance settings, students whose interpretive autonomy was supported from an early stage could set a mastery goal to convey intended interpretations and be the “true self [F]” during the performance. This experience itself satisfied them even when they received negative evaluations or made technical mistakes, motivating them to engage in the next performance.

In contrast, participants whose interpretive autonomy was hindered before college struggled to initiate SRL in performance. Before performances, they were pressured to reproduce interpretations, externally imposed by teachers, scores, norms, or ultimately themselves. During performances, they felt “detached [D]” from their own playing, and they took others’ negative feedback personally or could not appreciate positive feedback because they were dissatisfied with their own performance. These participants reported maladaptive states that are often associated with music performance anxiety (MPA), such as perceived performance impairment (Fehm and Schmidt, 2006; Murphy et al., 2025), playing-related physical injuries (Kenny and Ackermann, 2015; Amorim and Jorge, 2016), perfectionistic concern over mistakes (Liston et al., 2003; Yoshie and Shigemasu, 2007; Kobori et al., 2011; Dobos et al., 2019), the use of alcohol and drugs (Kenny et al., 2014; Hernández et al., 2018; Burin et al., 2019; Lupiáñez et al., 2022), and intentions to leave a music career (Hernández et al., 2018; Wang and Yang, 2024; Casanova et al., 2025).

The current and past studies suggest that promoting interpretive autonomy helps musicians self-regulate themselves in performance. Chen (2023) claimed that offering courses on musical interpretation led more students to report experiencing flow in performance. Others have shown that the improvisatory approach is effective in releasing anxiety. Learning and performing improvisation reduced music performance anxiety among young piano students (Allen, 2013), and professional musicians reported feeling less self-critical when adopting the interpretive approach than when they reproduced a prepared interpretation (Hill, 2017; Dolan et al., 2018). This leads us to the next discussion point―how can we nurture students’ interpretive autonomy?

### 4.2 How can we nurture music students’ interpretive autonomy?

#### 4.2.1 Factors behind need-thwarting learning experiences

Let us reflect on need-thwarting learning experiences first; why do they exist in the first place? Notably, six participants recognized the classical music field as need-thwarting; they felt interpretive autonomy was threatened by social pressure to conform to normative interpretations. Some participants were constantly over-challenged, forced, and rejected in musical interpretation from childhood to conform to those norms, which harmed their professional development and well-being. This paradox of the classical music industry and professional training, which is supposed to celebrate and nurture independent artists, is also observed in Wagner (2015). She found that students must conform to teachers’ expectations from the first stage, and “too much personality” is seen as “an obstacle to education” (p. 107). However, students were also expected to demonstrate artistic personality on stage as soloists, and many obedient students struggled to transform themselves into a “true artistic personality” in adulthood (p. 208).

Teachers were especially powerful in hindering interpretive autonomy, as they could control students’ behaviors in practice and performances. Need-thwarting teachers’ behaviors aligned with cautioned strategies in prior studies, such as an exclusive focus on technique (Rostvall and West, 2003; Young et al., 2003; Karlsson and Juslin, 2008; Gaunt, 2010; Holmgren, 2022), enforcement of certain interpretive ideas while rejecting students’ ideas (Persson, 1996; Silverman, 2008), and little demonstration (Rostvall and West, 2003; Burwell, 2021). They also added others, such as monotonous students’ playing styles and competition in a studio.

Importantly, need-thwarting experiences were often provided with good intentions to advance students’ careers. Some teachers criticized students’ interpretive ideas as violating the composer’s intentions, but they seemed more concerned with students’ short-term achievements which depended on other experts’ evaluations. Persson (1996) observed that an authoritarian teacher felt responsible for teaching the duty of “commitment to others’ expectations” and assessed students’ performances based on “the consensus” of good performances among other professors (pp. 41, 43). In competitions, atypical interpretations or prioritization of emotional investment over technical perfection result in elimination, but competitions offer a “unique opportunity to ‘build a career’” (McCormick, 2015; Wagner, 2015, p. 70). Finally, parents who assisted their children excessively wished to provide the best professional training. They selected teachers based on reputation―even though it did not guarantee that students’ interpretive autonomy would be supported―hoping for the career success of their child. Participants who felt incompetent, limited, and rejected in learning interpretation showed appreciation for their teachers and parents, understanding that the educators did what they felt “best [G]” for the participants.

Yet the more educators impose interpretive norms to prepare students for career success, the earlier and more intensely students’ interpretive autonomy may be suppressed. The more students are psychologically controlled, the more that they may pursue conformity out of anxiety or even stop performing professionally, reinforcing interpretive norms within the field and perpetuating the cycle.

#### 4.2.2 Providing need-supportive learning experiences

The study also provides hope, as interpretive autonomy could be promoted by need-supportive learning experiences.^4^

Teachers were, again, powerful in nurturing students’ interpretive autonomy. Having a teacher who always accepted their interpretations encouraged students to convey personal interpretations even in a challenging environment. Need-supportive teaching strategies aligned with those presented in Nerland’s (2007) study. A wind instrument teacher provided historical recordings and books, encouraged students to have original ideas, promoted peer collaboration, and allowed students to take lessons with others. This was to make the discourse of authenticity and instrumental traditions open and transparent so that students could “renew” them in a personal way (p. 410). Other effective strategies aligned with previous studies, such as open-ended questions (Meissner, 2017, 2021; Meissner and Timmers, 2019, 2020; Meissner et al., 2021), illustrative metaphors^5^ (Lindström et al., 2003; Schippers, 2006; Woody, 2006), and self-selected repertoires (Renwick and McPherson, 2002). Warm personality and encouragement were also appreciated, and most participants referred to their teachers as parental figures, such as “mom [A]” and “godfather [C].”

Since participants shared difficulties in finding or changing teachers, an issue that is not uncommon (Gaunt, 2011; Wagner, 2015), we now turn to experiences outside of lessons. Learning a wide variety of music—such as contemporary, cross-genre, free-style improvisation, or theatrical acting—in a classroom was also perceived as need-supportive, as it lets students explore expressive possibilities beyond norms within the field (Varvarigou, 2017; Hill, 2018). Additionally, performances at non-traditional venues, such as senior centers, were perceived as need-supportive “because there [was] no pressure from the audience [A],” in line with Paolantonio et al. (2022). Working with living composers was also beneficial because students could engage in open discussions on interpretation, fostering “creative collaboration” (Clarke et al., 2005, p. 44). Lastly, listening to a wide variety of recordings helped students explore interpretive ideas (Volioti and Williamon, 2021). Many participants appreciated Patricia Kopatchinskaja whose interpretations often sparked controversies among audiences (Leech-Wilkinson, 2020b). She “explained everything so clearly in her own way [F],” was “creative [C],” and made “us rethink what truth is if there’s any such thing in music [D].” G recalled that G would have “rejected all of that and been quite repulsed by it almost” when G was in college, then G continued:

“But actually, I think that she’s tapping into something very important for young people to see … [that] it’s okay to take risks on stage. … Stop thinking about perfectionism and impressing other people. And let us really try and go back to being master interpreters and creative artists rather than being slaves to the competition machine.”

We can also consider how need-thwarting learning environments could become more supportive. Parents may leave a space for their children for free self-exploration while providing necessary resources and support. Since studios are often isolated, conservatories may offer opportunities for instrumental teachers to self-reflect and collaborate with other teachers (Burwell et al., 2019). Institutions can also prepare courses for students to explore various genres of music-making, conduct workshops in a local community, and engage in multidisciplinary collaboration (Hill, 2018). Finally, competitions may have distinctive aims and juries rather than them being “almost always the same [F],” since competitions can “also be artsy … [having] their own identity [F].” They may also help winners with their careers so that they are not “completely forgotten [G].” Parents, conservatories, and competitions would contribute to students’ development significantly when they support their interpretive autonomy.

### 4.3 Limitation

While this study is grounded on valuable data from elite music students, there are limitations due to the qualitative method. Data relied on participants’ self-reports including those regarding interpretive autonomy and norms. Future studies may analyze other forms of data to investigate holistically. While case study research permits analytic generalizations, more empirical studies are needed to validate the plausibility of the model of *Werktreue* internalization. Additionally, no participants had need-thwarting learning experiences throughout their education, thus its effects remain unknown. Lastly, all the participants were in their 20s, and how the observed long-term effects may stay or diminish is unknown.

## 5 Conclusion

Having autonomy in interpretation―freely exploring and deciding what message to convey to audiences through performance―was a driving force for classical musicians to strive for excellence. Since interpretation is an essential part of music-making where musicians bring their own “voice,” interpretive autonomy allowed musicians to be their “true selves” even when they perceived the classical music field as over-challenging, controlling, and rejecting. In contrast, musicians with hindered interpretive autonomy perceived classical music performance as a harsh “battle [G],” where they compete against one another to reproduce an expected performance as flawlessly as possible. Musicians’ perceptions of the professional world were greatly shaped by early learning experiences; “casual [A, C, F]” exploration laid a strong foundation for interpretive autonomy, while the enforcement of normative interpretations suppressed their voices.

Autonomous interpreters would not only exhibit strong resilience when the classical music field poses challenges but could also contribute to transforming the field into a more supportive environment. When musicians creatively bring personal interpretations, they enhance interpretive diversity, fostering a healthier artistic ecosystem. To support musicians’ optimal professional development and the evolution of the classical music field, parents, teachers, gatekeepers, performers, and researchers play a crucial role in reconsidering professional training from an early stage.

## Data Availability

The raw data supporting the conclusions of this article will be made available by the authors, without undue reservation.

## Footnotes

^1^Self-regulated learning is a cyclical process consisting of three phases: forethought, performance, and self-reflection. In the forethought phase, self-regulated learners set specific goals and plans while motivating themselves for the task. During the performance phase, they employ various learning strategies while monitoring their effectiveness. Finally, in the self-reflection phase, they self-evaluate their learning performance and attribute errors appropriately. Learners then motivate themselves to engage in the next cycle, setting new goals and plans (Zimmerman, 2002).

^2^The term “non-need-thwarting learning experiences” refers to experiences that were perceived as need-thwarting by other participants yet were not perceived as such by the participant.

^3^This is an example of non-need-thwarting learning experience.

^4^The long-term effects were observed only at college and post-college level; E, D, G continue to engage in performing, teaching, and deepening their knowledge with their passion, which may mitigate fear and anxiety further, leading them to fully embrace the joy of music-making.

^5^Note that when metaphors are difficult to understand, they can be frustrating as illustrated in D’s case.
